# Cell-free and cell-bound circulating DNA in breast tumours: DNA quantification and analysis of tumour-related gene methylation

**DOI:** 10.1038/sj.bjc.6603117

**Published:** 2006-04-25

**Authors:** T E Skvortsova, E Y Rykova, S N Tamkovich, O E Bryzgunova, A V Starikov, N P Kuznetsova, V V Vlassov, P P Laktionov

**Affiliations:** 1 Institute of Chemical Biology and Fundamental Medicine, SD RAS, 8, Lavrentiev ave., Novosibirsk 630090, Russia; 2 National Novosibirsk Regional Oncologic Dispensary, 2, Plahotnogo str., Novosibirsk, Russia

**Keywords:** breast cancer, diagnostics, circulating DNA, methylation-specific PCR, DNA quantification

## Abstract

Tumour development is characterised by the increased circulating DNA (cirDNA) concentration and by tumour-related changes in blood plasma DNA. Concentration of cirDNA and methylation of RAR*β*2, RASSF1A and HIC-1 gene promoters were investigated in cell-free and cell-surface-bound fractions from healthy donors, patients with breast cancer, and patients with breast fibroadenoma. Tumour development was shown to lead to significant changes in the distribution of cirDNA between cell-free and cell-surface-bound fractions. Analysis of RAR*β*2 and RASSF1A methylation in the total cirDNA provides 95% diagnostic coverage in breast cancer patients, 60% in patients with benign lesions, and is without false-positive results in healthy women. Results of the study indicate that methylation-specific PCR of RAR*β*2 and RASSF1A genes based on the total cirDNA combined with the quantitative analysis of cirDNA distribution between cell-bound and cell-free fractions in blood provide the sensitive and accurate detection and discrimination of malignant and benign breast tumours.

In the last years attempts have been made to develop noninvasive tests for early cancer diagnostics based on analysis of extracellular DNA circulating (cirDNA) in the blood. The increased plasma cirDNA concentration itself can be an indication of tumour development (Anker, 2000). However, this test did not promise to be specific, as far as the comparable increase of DNA concentration in plasma was found in patients with other disorders as systemic lupus erythematosus, rheumatoid arthritis, glomerulonephritis, pancreatitis, hepatitis, etc (Anker, 2000).

Numerous studies have demonstrated tumour-specific alterations, such as aberrant promoter hypermethylation in cirDNA recovered from plasma of patients with different malignancies, and the absence of methylated DNA in healthy persons (Silva *et al*, 2002). Changes in the status of DNA methylation represent one of the most common molecular alterations in human neoplasia (Egger *et al*, 2004), including breast cancer (Widschwendter and Jones, 2002). These epigenetic alterations induce neoplastic process by transcriptional silencing of tumour suppressor gene expression and are responsible for initial steps of induction of tumour cell proliferation (Jones and Baylin, 2002). Therefore, analysis of gene methylation patterns in tissues could be of profound significance in the early detection of cancer (Esteller *et al*, 2001). Expression of more than 40 genes was found to be lost in breast cancer because of promoter hypermethylation (Jones and Baylin, 2002; Widschwendter and Jones, 2002), including RASSF1A, RAR*β*2, HIC-1 genes. However, the methylated markers are detected with twice lower frequency in plasma samples compared with the frequency of their finding in the tissues of cancer patient (Shao and Nguyen, 2002; Anker P *et al*, 2003). According to our recent observations, extracellular nucleic acids circulate in blood not only in plasma but are also bound to the surface of blood cells (Rykova *et al*, 2004). In pilot experiments we evaluated the cell-surface-bound cirDNA as a source of material for the early breast cancer diagnostics based on methylation-specific PCR (MSP) (Rykova *et al*, 2004). In the present study we investigated the methylation of HIC-1, RASSF1A and RAR*β*2 genes promoters in cirDNA derived from plasma and cell-surface-bound fractions of patients with breast tumours.

Results of the study evidence that the analysis of the cell-surface-bound cirDNA along with cirDNA from plasma fraction could considerably increase reliability of noninvasive tests for tumour development based on MSP.

## MATERIALS AND METHODS

### Blood samples

Blood samples of previously untreated breast cancer and nonmalignant patients were obtained from Novosibirsk Regional Oncology Dispensary. The Ethics Committee in Dispensary approved specimen collection procedures. Tumour staging was performed according to the TNM classification. Blood samples of healthy donors, selected for blood transfusion, were obtained from Novosibirsk Central Clinical Hospital. Blood (8 ml) was collected into the tubes containing 2 ml of sterile phosphate-buffered saline (PBS) with 50 mM EDTA. Blood was fractionated into plasma, leucocytes and erythrocytes, and cell-surface-bound DNA fractions were obtained as described earlier (Rykova *et al*, 2004).

### DNA extraction and concentration measurement

DNA was extracted from 1 ml of plasma samples, 2 ml of PBS/EDTA samples and 1 ml of trypsin samples by glass milk-based protocol providing quantitative isolation of DNA (Tamkovich *et al*, 2004). Concentration of DNA was measured using Hoechst 33258 assay (Labarca and Paigen, 1980). Detection limit for DNA calculated to the initial blood volume was 8 ng ml^−1^ in plasma, 20 ng ml^−1^ in trypsin eluates and 40 ng ml^−1^ in PBS/EDTA eluates.

### Methylation-specific PCR

Aberrant methylation of RASSF1A, RAR*β*2 and HIC-1 gene promoters was determined by MSP according to modified protocol (Herman *et al*, 1996). DNA was extracted from 1 ml of plasma samples, 6 ml of PBS-EDTA samples and 1 ml of trypsin samples by glass milk-based protocol (Tamkovich *et al*, 2004). Isolated DNA was modified by sodium bisulfite, purified using glass milk protocol, eluted into 30 *μ*l of water and stored in aliquots at −40°C. A volume of 3 *μ*l of the bisulfite-modified DNA was used in MSP reaction. The primers used are listed in Table 1. Polymerase chain reaction products were visualised on 6% PAAG stained with ethidium bromide.

## RESULTS

**Figure 1:**
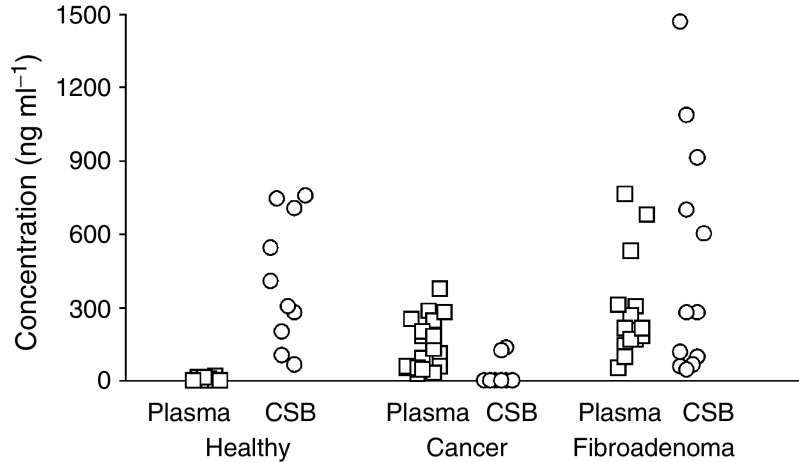
Concentrations of circulating DNA in plasma (*Plasma*) and cell-surface-bound DNA (*CSB*) in blood of healthy women (*Health*), breast cancer patients (*Cancer*) and patients with breast fibroadenoma (*Fibroadenoma*).

We analysed concentrations of cell-surface-bound and cell-free cirDNA in the blood samples of 20 breast cancer patients, 15 patients with nonmalignant tumour (fibroadenoma) and 10 healthy women. Analysis of the distribution of cirDNA in blood of breast cancer patients supports our earlier data (Laktionov *et al*, 2004) that the main part of the cirDNA in the blood of healthy donors is absorbed at the surface of blood cells (Figure 1). A strong decrease of the cell-surface-bound cirDNA amount, as well as an increase of cell-free cirDNA was found in cancer relative to health (Figure 1). In the blood of 95% of breast cancer patients, increased amounts of cirDNA were found in plasma and only negligible amounts of cell-surface-bound cirDNA were found in few patients of this group. Patients with fibroadenoma were characterised by increased level of circulating DNA in plasma similar to cancer patients, albeit cirDNA were found also at the surface of blood cells similar to what was found in healthy women (Figure 1).

Methylation of RASSF1A, RAR*β*2 and HIC-1 gene promoters were studied in healthy donors and patients with breast tumours. These genes had been reported to be frequently methylated in breast cancer (Esteller *et al*, 2001; Jones and Baylin, 2002; Widschwendter and Jones, 2002). CirDNA isolated from the plasma of 10 healthy women were found to be negative for methylation forms of all three genes. Methylation of RAR*β*2 and RASSF1A promoters was not found in the cirDNA isolated from cell-surface-bound fractions, whereas five healthy women out of ten were positive for methylated HIC-1 in different cell-associated cirDNA fractions (Table 2). Methylated form of RASSF1A gene was detected with the same frequency (60%) in the cell-bound cirDNA from patients with cancer and nonmalignant breast tumours (Table 2). In contrast, methylated RAR*β*2 form was detected three times more frequently in breast cancer patients than in fibroadenoma patients: in 60% of cell-associated cirDNA in cancer and 20% in nonmalignant cases (Table 2). The methylation of all three genes was detected considerably more frequently in the cell-bound cirDNA than in the plasma cirDNA of the tumour-bearing patients (Table 2). The methylation pattern of the different genes differ among patients; some patients demonstrated simultaneous methylation of RAR*β*2 and RASSF1A, while some of them were positive only in either RAR*β*2 or RASSF1A regions. To summarise, two methylated markers RAR*β*2 and RASSF1A were not found in healthy women but were detected in 95% of the cancer patients and in 60% of the patients with fibroadenoma when total circulating DNA were used for MSP reaction (Table 2).

## DISCUSSION

CirDNA is present in blood plasma in health and is increased in cancer and other malignancies (Anker, 2000). These findings have attracted much attention to the potential use of elevated concentration of cirDNA as a tumour marker. It is known that nucleic acids can bind with cell-surface DNA-binding proteins (Bennett *et al*, 1985; Laktionov *et al*, 1999), as well as with phospholipids of cellular membrane through bivalent ions (Beliaev *et al*, 1988). These interactions together with large amount of cells in the bloodstream provide an opportunity for circulation of DNA in blood being absorbed at cell surface. Earlier we have found that a fraction of the bound nucleic acids could be detached from the cell surface by PBS/EDTA treatment suggesting involvement of bivalent ions in the complex formation. Further removal of the surface-bound cirDNA can be achieved by mild trypsin treatment, which destroys cell surface proteins binding with cirDNA. In accordance with previous data (Laktionov *et al*, 2004) results of the present study indicate that the main part of the cirDNA in the blood of healthy donors is mainly absorbed at the surface of blood cells (Table 2). Our data demonstrate redistribution of extracellular DNA in blood between cell surface and plasma in breast tumour-bearing patients, which suggest a possibility of the development of simple noninvasive tumour-screening assay.

Mechanisms regulating appearance and distribution of extracellular DNA in blood are not clear to date. In the bloodstream extracellular DNA are under the pressure of factors influencing its circulation and clearance, including hydrolysing enzymes. It is known that tumour invasion in cancer patients is accompanied by increased level of proteases (Farias *et al*, 2000). Damage of the cell-surface DNA-binding proteins by these enzymes can result in detachment of cell-bound DNA. Cancer development is characterised by the change of DNase activity in blood plasma. It was shown recently, that the decreased DNase activity in plasma of patients with gastrointestinal cancer correlates with the increased integrity of cirDNA and their concentration in blood plasma (Tamkovich *et al*, 2006). Other factors should be further investigated, which could influence extracellular DNA distribution in the circulation in health and in the process of tumour development.

Tumour-related methylated forms of three genes (RAR*β*2, RASSF1A and HIC-1) were much more frequently evaluated in the cell-surface-bound cirDNA compared with cell-free cirDNA in tumour-bearing patients (Table 2). The data obtained clearly indicate that cell-surface-bound DNA provides valuable source of material for MSP diagnostics along with cirDNA isolated from the plasma. According to our data RAR*β*2 gene methylation possesses the most cancer-specific propensity. In contrast, methylation of HIC-1 promoter region, which was examined here, is not tumour specific.

Analysis of methylated DNA sequences in the cirDNA demonstrated no regularities in their fraction distribution (Table 2). Phosphate-buffered saline and trypsin treatments both provide cell-bound DNA, which should be used combined as a template for the MSP-based assay. There is no significant difference in the methylation frequencies for the leucocyte-bound cirDNA compared with erythrocyte-bound cirDNA, when any of three genes was analysed. However, as long as there are 1000 times less leucocytes than erythrocytes in blood, we propose that specific content of methylated DNA at the surface of a single leucocyte is higher than at the surface of a single erythrocyte.

Whatever might be the mechanism for tumour-related DNA binding to blood cells, we can now ask whether this phenomenon has potentially important implications in cancer patients. Experiments with human fibroblasts provided evidences that tumour DNA may be horizontally transferred by the uptake of apoptotic bodies (Holmgren *et al*, 1999). Garcia-Olmo *et al* (1999)suggested that metastases might develop as a result of transfection of susceptible cells in distant target organs with dominant oncogenes that circulate in plasma and are derived from the primary tumour. Monocyte cells from leucocyte fraction, which can migrate into the tissues from the blood vessels, could play the role of ‘carriers’ of the surface-bound tumour DNA to healthy cells.

Our data indicate the presence of methylated RASSF1A and RAR*β*2 in the cirDNA of all the detected patients bearing the Ist tumour stage. This sounds optimistic for the development of tumour-specific screening test, taking into account the early stage of these changes to occur. To summarise, assay based on the methylated RAR*β*2 or RASSF1A detection in the total circulating DNA in blood provides 95% sensitivity for breast cancer detection. Additional quantitative analysis of the distribution of extracellular DNA between cell-bound and cell-free fractions in blood is helpful to differentiate healthy women, patients with breast fibroadenoma and breast cancer patients.

## Figures and Tables

**Table 1:** Sequences of primers used in MSP

**Gene**	**Primer sequence (5′–3′)**	**Genbank accession no.**	**Amplicon location (Genebank numbering)**	**T (in °C)**	**Length (in bp)**
RASSF1A	Mf: GGG TTT TGC GAG AGC GCG				169
	Mr: GCT AAC AAA CGC GAA CCG	AC002481	17881–18115	64	
					
	Uf: GGT TTT GTG AGA GTG TGT TTAG			59	169
	Ur: CAC TAA CAA ACA CAA ACC AAG				
					
RAR*β*2	Mf: GCT TAG TAG TTC GGG TAG GGT TTA TC				235
	Mr: CCG AAT CCT ACC CCG ACG	X56849	773–1007	64	
					
	Uf: TTA GTA GTT TGG GTA GGG TTT ATT			55	233
	Ur: CCA AAT CCT ACC CCA ACA				
					
HIC-1	Mf: TCG GTT TTC GCG TTT TGT TCG T				95
	Mr: AAC CGA AAA CTA TCA ACC CTC G	L41919	26–120	64	
					
	Uf: TTG GGT TTG GTT TTT GTG TTT TG			64	118
	Ur: CAC CCT AAC ACC ACC CTA AC				

**Table 2:** Frequency of methylation of RASSF1A, RAR*β* and HIC-1 genes in plasma and cell-surface-bound DNA in blood of breast cancer patients, patients with fibroadenoma and healthy donors

			**Erythrocytes**		**Leucocytes**			
**Diagnosis**	**Gene**	**Plasma**	**PBS eluate**	**Trypsin eluate**	**PBS eluate**	**Trypsin eluate**	**Total cell-surf- bound**	**Plasma and total cell-surf-bound**
Breast cancer (*n*=20)	HIC-1	55*	35	45	70	45	90	90
	RASSF1A	15	35	0	35	30	65	75
	RAR*β*2	15	5	25	20	15	60	65
	RASSF1A or RAR*β*2	30**						95
								
Fibroadenoma (*n*=15)	HIC-1	40	27	73	67	53	93	93
	RASSF1A	7	27	33	40	27	53	53
	RAR*β*2	13	0	7	0	13	20	33
	RASSF1A or RAR*β*2	13						60
								
Healthy donors (*n*=10)	HIC-1	0	10	10	50	20	50	50
	RASSF1A	0	0	0	0	0	0	0
	RAR*β*2	0	0	0	0	0	0	0
	RASSF1A or RAR*β*2	0						0

* Percentage of cases with RASSF1A, RAR*β*2, HIC-1 hypermethylation in plasma DNA, cell-surface bound DNA and total circulating DNA.

** Percentage of cases with RASSF1A or RAR*β*2 hypermethylation in plasma DNA and total circulating DNA.
